# Surgical treatment of locally advanced thyroid cancer

**DOI:** 10.1515/iss-2020-0012

**Published:** 2020-09-11

**Authors:** Rudolf Roka

**Affiliations:** Acute and Endocrine Surgery, Sigmund Freud Private University Vienna, Wien, Austria

**Keywords:** advanced thyroid cancer, multivisceral resection, reconstruction

## Abstract

Operations in this area are demanding and require special experience in endocrine, thoracic and vascular surgery, an experienced anaesthesiologist, as well as the interdisciplinary cooperation with other medical specialists (nuclear medicine, oncology, radiology, otolaryngology). A reliable system of surgical guidelines has been developed from a few individual publications with special impact.

## Introduction

Although differentiated thyroid cancer (DTC) in locally limited tumours has an excellent prognosis with an up to 100% survival rate, failure to control local disease due to invasive cancer is one of the main risk factors for recurrence or mortality [1], [2]. Direct tumour extension with invasion of the surrounding tissue is seen in up to 22% of patients [3], [4], [5] and cervicovisceral invasion in 4–10.9% [6], [7]. The percentage of patients dying from local complications (airway obstruction, vascular invasion, haemorrhage, malnutrition) has been reported to be 36–47% or even higher. The structures most commonly affected by direct invasion are the strap muscles, larynx, trachea, oesophagus and the recurrent laryngeal nerve. In the lateral neck and the mediastinum, lymph node metastases may invade the large veins, bone, the sternocleidomastoid muscle, the pleura, the lung and rarely also the carotid artery.

Advanced tumour stage with infiltration of the surrounding structures and organs is the strongest prognostic factor. The particular challenge of resections in the pharyngo-laryngo-tracheal region is preserving a complex system. Preservation and reconstruction are an important goal and difficult to achieve. On the other hand, it is necessary to proceed radically to avoid recurrence and its risks. Every surgical concept seems to be a balancing act of meeting oncological needs and achieving a functional outcome.

Local invasion is, however, only one of the problems seen in such patients. A high rate of distant metastases, advanced age, preoperative radiotreatment, repeated surgery and – most relevantly – aggressive tumour biology (tall-cell, columnar-lined, insular, poorly differentiated tumours and Hürthle cell tumours) may all result in therapeutic failure.

The morphological correlation has been well described but has not yet been classified into a general standardized grading system.

As tumour characteristics and patients’ conditions vary widely and surgeons tend to disagree on optimal treatment options, patient groups are small and cannot be compared. As a result, there are only retrospective studies of diverse patient groups that yield limited evidence. The views expressed in the literature can essentially be divided into two groups. Some of these studies are summarized in Table 1.

**Table 1:** Different surgical approach of specialized authors [1, 8–27].

Author	N	Circumferential sleeve – resect.	Partial resection, fenestration	Shaving, mainly Shin 1	Adjuvant treatment
Price 2008	Review	x	**x**	**x**	**x**
Ozaki 1995	21	x			
Shindo 2014 AHNS Consensus	Review AHNS Consens.	x	**x**	**x**	**x**
Kim 2016	65	x		**x**	
Honings 2010	Review	x			
Gillenwater 1999	Review	x	**x**		
Grillo 1992	34	x			
Tanaka 1999		x			
Braukhoff 2009	Technique	x	**x**	**x**	
Ishihara 1991	60	x			
Avenia 2017	37	x			
Mc Caffrey 1990	66	x	**x**	**x**	**x**
Gaissert 2007	82	x			
Segal 2006	49	x	**x**	**x**	**x**
Nishida 1997	117	x	**x**	**x**	**x**
Ito 2009	107		**x**	**x**	
Wada 2006	64	x	**x**	**x**	
Tsai 2005	33	x	**x**	**x**	
Hartl 2014	26	x			
Kebebew 2003	Review	x	**x**	**x**	**x**
Roka 2014	39	x	**x**	**x**	**x**

The development and availability of diagnostics but probably also the education and attention of the patients mean that grotesquely advanced cases and a high rate of metastases on first presentation are hardly ever observed any more in contrast to previous decades.

### Symptoms

Specific symptoms are hoarseness and dyspnoea, a growing coarse tumour, and in advanced cases pain, swallowing disorders, Horner’s syndrome and signs of skin infiltration, especially in aggressive forms.

Intraluminal lesions in the trachea or oesophagus may lead to dangerous bleeding. On the other hand, superficial infiltration of the cartilage skeleton often remains without symptoms for a long time.

### Preoperative diagnosis

Especially the superficial infiltration of the pharyngo-laryngo-tracheal system is difficult to determine. In particularly experienced hands, a correct diagnosis with ultrasound has been described in up to 85% of cases [28]. If there is clinical suspicion or suspicious proximity in ultrasound, the diagnosis can be confirmed by CT. However, sensitivity is low [29]. The administration of a contrast agent, which delays radioiodine therapy by weeks, should be agreed on an interdisciplinary basis.

The endoscopic investigation of the larynx, primarily as video laryngoscopy, is essential as before any thyroid intervention [8].

Tracheoscopy and oesophagoscopy are performed selectively if endoluminal penetration of a tumour is suspected [8].

### Pathological assessment of radicality

With large resection areas and occasionally also with cartilage tissue, it is difficult to do a complete pathological (R0 or R1) evaluation of the excision margins. The focus is therefore also on the distinction from R2 resections (leaving gross tumour behind). Prognostic differences between R0 and R1 are often assessed as small [30], [31].

### Infiltration of the recurrent laryngeal nerve

One of the typical situations is infiltration of the recurrent laryngeal nerve, occasionally by small extracapsularly developed papillary carcinomas. In view of the functional consequences, a concept [32] was developed as a compromise which allows minimal tumour residue on the nerve after neurolysis – but only if the function of the nerve is preserved (Table 2). The procedure is usually done even more generously regarding the residual tumour if there is already recurrent paresis on the contralateral side, and the danger of bilateral paresis is imminent. Neurolysis of a paretic nerve does not yield any success unless exact inspection suggests that the paresis was merely caused by pressure and not by tumour infiltration [33].

**Table 2:** Algorithm for infiltration of the recurrent laryngeal nerve.

**Histological confirmed tumour infiltration**
Preoperative paralysis on the tumour side: Resect RLN, poss. laryngeal nerve reinnervation
Normal function on both sides: shaving with removal of all gross disease and adjuvant treatment
Normal function on the tumour side and contralateral paralysis: shaving, removal of the tumour as much as possible and adjuvant treatment
Reconstruction of the resected nerve may be advantageous

After prolonged neurolysis, paresis is to be expected in up to one third of cases [34]. Continuous neuromonitoring is therefore imperative in these cases.

While reinervation of the recurrent laryngeal nerve by direct suture or transplant cannot restore the original function, it can significantly improve postoperative voice quality [11].

### Infiltration of the pharynx und oesophagus

Isolated evidence of tumour in the oesophagus is rare. Due to its wall structure and strong mucosa, lateral infiltrations can be removed relatively easily by resection of the affected muscle layers. Circular resection is only possible over a very short distance and is difficult because the cervical oesophagus can only be mobilized for tensionless anastomosis to a very limited extent [34].

In contrast to the oesophagus, the wall of the pharynx is thin and vulnerable. Direct suture closure of larger defects, especially when tension is applied to the suture line, is particularly susceptible to dehiscence. In such cases, closure with a revascularized small intestine patch is the best option. An alternative is closure with a sufficiently large pectoralis major flap.

### Surgical therapy of the trachea

**Figure 1:**
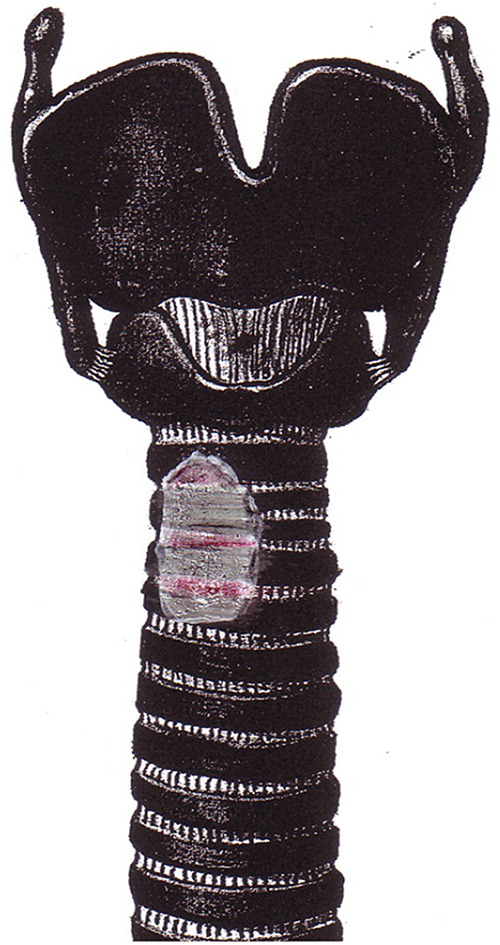
Tracheal shaving.

**Figure 2:**
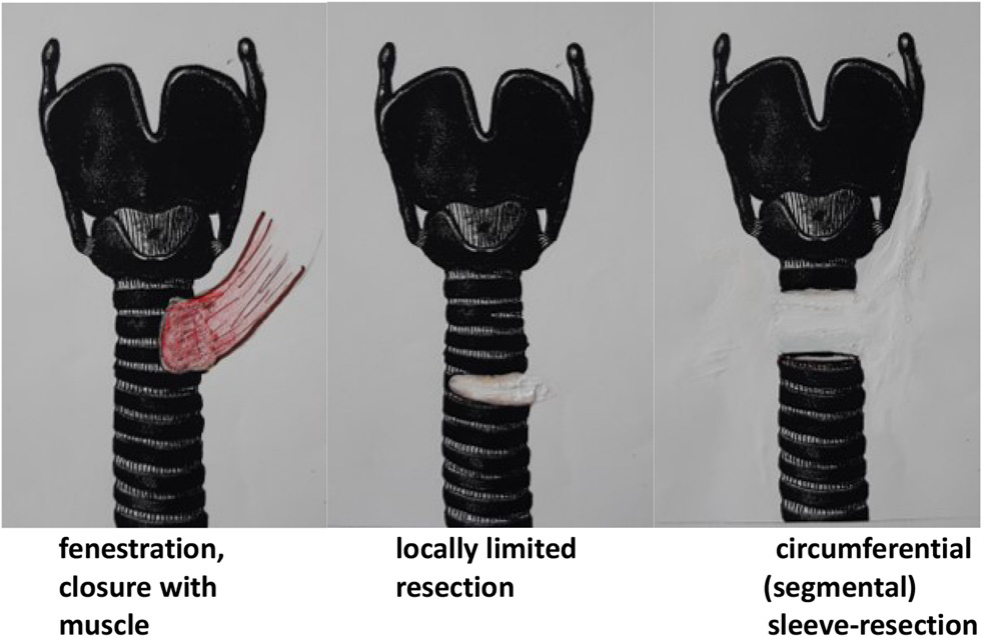
Resections on the trachea.

Most authors advocate a selective approach, depending on preoperative diagnostics and intraoperative assessment, by means of shaving (Figure 1), partial resection or transverse circumferential (sleeve) resection of the trachea (Figure 2).

**Figure 3:**
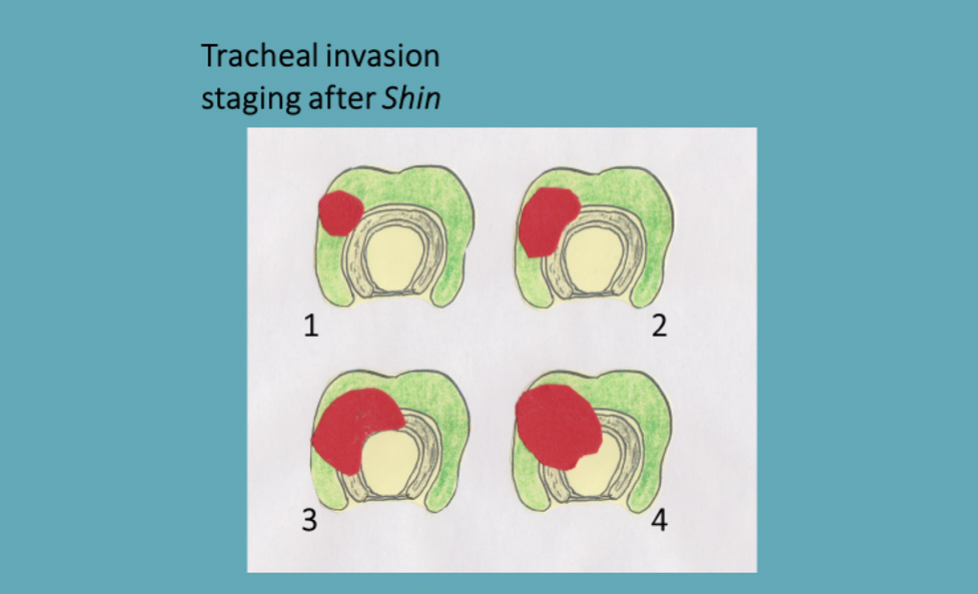
Tracheal invasion staging after Shin.

Staging of tracheal invasion was presented by Shin et al. [35] and comprises four stages (Table 3, Figure 3 ). It largely corresponds to a previous study by Ozaki et al. [12]. In a detailed histopathological workup of 22 cases, Ozaki et al. [12] were able to establish that the external macroscopic appearance does not correspond to the actual extent of infiltration within the tracheal wall: If the tumour reaches into the submucosal space, the circumferential extension is in most cases much more extensive than at the adventitia outside. These investigations suggest an insufficient or uncertain macroscopic assessment and support full-thickness resection as soon as the tumour has reached the perichondrium.

Nevertheless, shaving is considered one of the established methods but only up to Shin stage I. Excellent long-term results have been reported for differentiated tumours with subsequent radioiodine therapy. In contrast, shaving is not to be used for aggressive, poorly differentiated tumours [36].

Among the full-thickness procedures, the complete transverse resection of the trachea is the oncologically safest but also the most complex procedure [26]. In this procedure, special attention must be paid to the blood supply to the tracheal edges in the area of the anastomosis. Blood supply is fed by the network of the inferior thyroid artery, which may be compromised by tumour penetration or extensive preparations in the central compartment. Special attention should also be paid to the anatomical proximity of the recurrent laryngeal nerve. If necessary, anastomotic tension of the trachea must be reduced by special measures (suprahyoid release). Alternatives to full-thickness resections are fenestration and partial resection [27] (Figure 2). These procedures are, however, only possible up to about one third of the tracheal circumference without loss of stability and often cannot cope with the circumferential spread of the tumour [32].

A further important argument in favour of primary transverse resection of the trachea is found in a study by Gaissert et al. [21], who were able to demonstrate that resection as a secondary intervention in the case of local recurrence (e.g. after shaving) is associated with a significantly worse prognosis than primary resection. This is a point that should be taken into account especially with younger patients.

In principle, oncologically stable haematogenic metastases in differentiated carcinomas and possible R0 resection are not a contraindication against transverse resection of the trachea [8], [15], [37].

### Resections at the larynx

**Figure 4:**
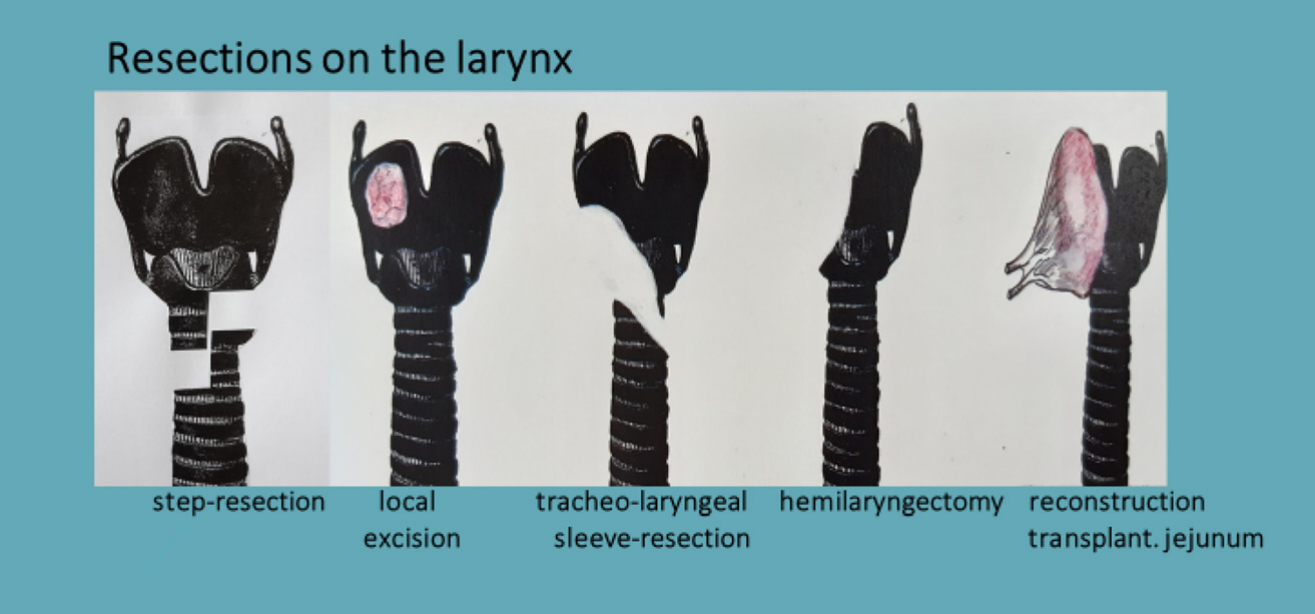
Resections of the larynx.

The cricoid ring and also parts of the thyroid cartilage can be resected in limited anterolateral areas without loss of stability. Small lesions are then usually covered with a muscle flap. Extensive lesions require a crico-tracheal step or sleeve resection. Both measures are complex, and special attention must be paid to preserving the contralateral recurrent laryngeal nerve (Figure 4).

**Figure 5:**
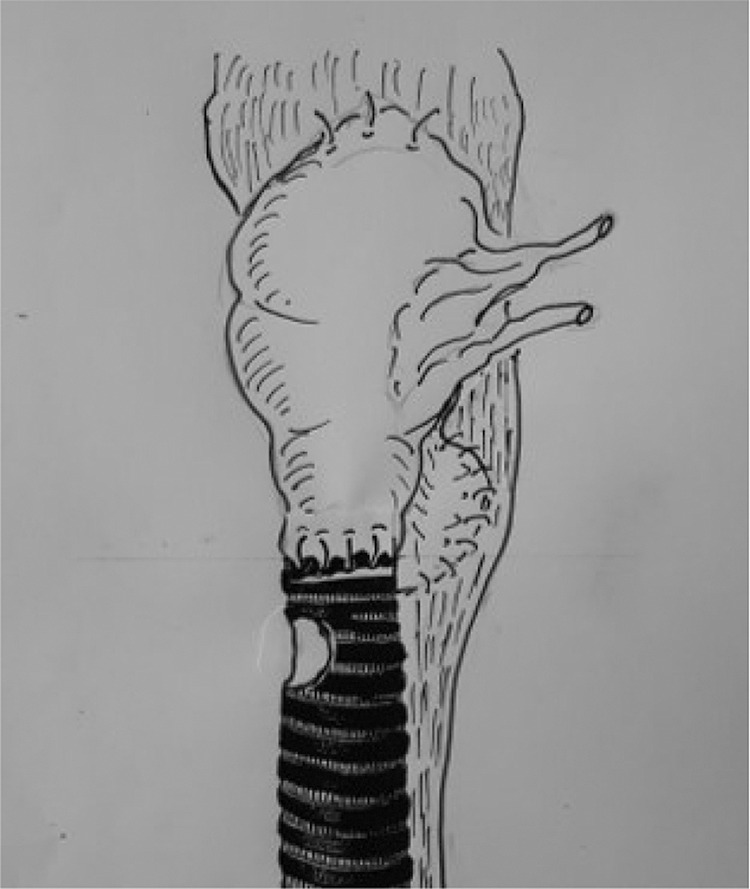
Jejunal interposition (tracheo-pharyngeal shunt) with closure of the pharyngeal defect after laryngectomy.

**Figure 6:**
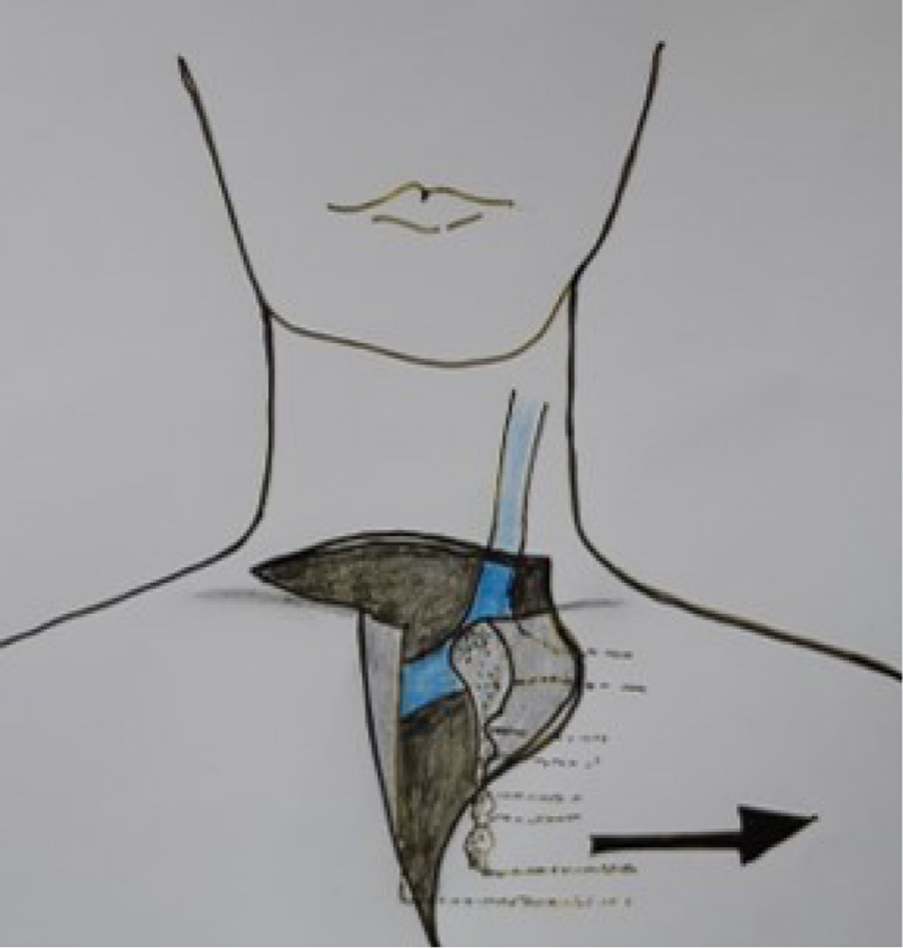
Partial sternotomy extending into the third intercostal space.

To cover larger defects, especially when parts of the pharynx are also involved, transplanting a revascularized small intestine patch is particularly suitable [38] (Figures 5 and 6).

### Laryngectomy

Complete removal of the larynx results in the greatest functional loss when operating on cross-organ thyroid carcinoma. The decision may only be made after interdisciplinary discussion of possible alternatives, such as partial resection or, in emergencies, simply tracheotomy or endoscopic methods for haemostasis.

The pharyngeal wound can be closed by direct suture or by means of a revascularized small intestine patch. Should this be necessary due to an extensive wall defect, A tracheo-pharyngeal shunt might also be considered: The interposition of a small intestinal loop with pharynx-directed peristalsis between the end of the trachea and the pharynx allows the closure of the defect and also acts as a neolarynx by using its own respiratory air when occluding a tracheostoma underneath. The inflated loop of the small intestine then acts as a kind of air chamber and the intestino-pharyngeal anastomosis as neoglottis [39]. This method was successfully used years ago in numerous cases of laryngeal carcinoma when laryngectomy was performed much more frequently (Figure 5).

**Figure 7:**
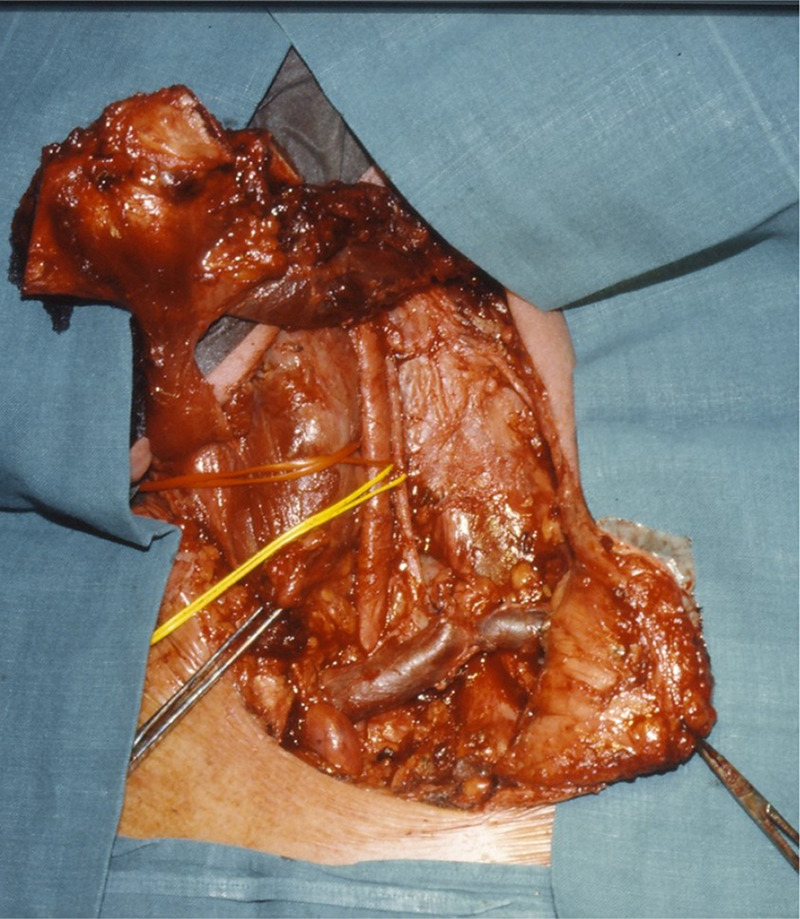
Approach after Killian.

An analysis of 10,215 patients [14] shows that in 5.8% of cases, invasion of the tracheal system occurs and is one of the most frequent causes of death. Recommendations and outcomes vary widely in the literature for various respective procedures. When choosing the appropriate procedure, the individual situation must be taken into account. Publications with special impact on the treatment are listed in Table 4.

**Table 3:** Staging system for tracheal invasion after Shin.

Stage 1: Tumour invades through the capsule of the thyroid gland but does not invade the external perichondrium.
Stage 2: Tumour invades the cartilage
Stage 3: Tumour extends into the lamina propria of the tracheal mucosa
Stage 4: Full-thickness invasion into the lumen

**Table 4:** Publications with special impact for the treatment [8–10, 36].

Author	N	Resected structures	Results	
Wang 2016	153	Trachea Larynx Oesophagus	5 year DSS	R0 94.4% no stat. R1 87.6% diff.
Hartl 2013	46	Trachea Larynx Oesophagus	5 year DSS	M0/R0 95% M0/R1 84% M1/R0 68% M1/R1 34%
Gaissert 2007	82	Trachea Larynx	DFS 10 years	Sleeve resection early 67% delayed 7%
Ishihara 1991	60	Trachea	10 years OS	Complete resection 78.1% Incomplete resection 24.3%

Large pharyngeal defect; DSS, ; OS, overall survival.

### Mediastino-thoracic access

The standard access for mediastinal spread is partial or total sternotomy.

Partial sternotomy, however, offers a limited view of the lateral sections of the mediastinum. This can be improved by extending the access by transversely cutting the sternum and thus reaching the intercostal space on the affected side [40] (Figure 6).

An alternative is also the access according to Killian [41] (Figure 7) here, the resection of the sternoclavicular joint is performed including the insertion of the first rib. The resected tissue remains attached to the sternocleidomastoid muscle, supplied with blood, and is then repositioned.

Oncological resections of proximal parts of the sternum, including the sternoclavicular joint, remain relatively asymptomatic due to the stable connective tissue anchoring.

### Resection of the cervico-mediastinal vascular system

Resections on the large arteries are rarely considered since there is seldom a meaningful indication as carcinomas are extensive and almost always undifferentiated [34].

The large veins (internal jugular vein, subclavian vein, innominate vein), on the other hand, are frequently affected. Unilateral resection of the internal jugular vein is unproblematic. If bilateral resection is required, it is often necessary to wait several weeks between the two procedures. In case of tumour closure of the innominate vein, reconstruction is not necessary since collateralization occurs via the axillary venous system.

Follicular carcinoma tends to bring about venous infiltration and may occasionally grow in a freely floating manner intravascularly as a tumour thrombus up to the right atrium. Due to the risk of rupture with fatal consequences, careful removal is imperative after securing vena cava flow.

### Visceral resections in case of haematogenic metastases

In differentiated carcinomas, cervicovisceral resections are not contraindicated, at least if there is macroscopic completeness (R1, R0) [8]. In poorly differentiated or undifferentiated tumours, however, R2 resections should be done only as exceptions and in emergency situations (such as persistent bleeding or respiratory problems) [8], [32].

Due to the very limited life expectancy of patients, most of whom are of advanced age, and the resulting high risk of surgery, measures such as tracheostomy or stents are therefore preferable [42].

### Radioiodine therapy and external radiotherapy

Most authors, especially those whose therapeutic concepts include shaving at the laryngo-tracheal area or incomplete resections, advocate postoperative radioiodine therapy for differentiated tumours [43] and external high-voltage therapy as supplementary ablation for aggressive forms [44], [45].

Kebebew and Clark [1] and Nixon et al. [46] are sceptical about the sustainable value of these measures, while Gillenwater and Goepfert [15] believe that they can prevent local recurrences. So far, there is no high degree of evidence, however, to support these measures.

It is interesting, however, to note a case report by Shingu et al. [47], which provides evidence of the preoperative effect of radioiodine therapy leading to subsequent resectability of an initially inoperable tumour.

### Outlook

The uncomplicated thyroid carcinoma, the excellent prognosis and low morbidity in endocrine centres of excellence make this one of the most satisfying areas of a surgeon’s life. With locally advanced, metastasized and histologically aggressive tumours, on the other hand, improvements in surgical outcomes will probably also depend very much on the success of molecular biology research [37].

Currently, inhibition of tumour progression has been achieved by the use of tyrosine kinase inhibitors [48], [49], [50]. Another promising development is the reinduction of sodium iodine symporter (NIS)-mediated radioiodine storage with the member of the mitogen-activated protein kinase (MEK) 1/2 inhibitor selumetinib.

Molecular subtyping of differentiated thyroid carcinomas is also progressing with the determination of B-Raf mutations and RET-PT rearrangements in papillary and Ras mutations in follicular carcinoma – first promising steps towards targeted drug therapy [51].

The future will show to what extent it will be possible to reduce surgical radicality in visceral problem areas and whether it will be possible to do without surgical ablation in the highly aggressive forms – as has been achieved in individual cases.

At present, however, success is primarily due to the cooperation of experienced endocrine surgeons with the other specialists of the interdisciplinary team and the observance of the guidelines based on thorough analysis of the literature.

## Summary

Operations in this area are demanding and require special experience in endocrine, thoracic and vascular surgery, an experienced anaesthesiologist, as well as the interdisciplinary cooperation with other medical specialists (nuclear medicine, oncology, radiology, otolaryngology).

A reliable system of surgical guidelines has been developed from a few individual publications with special impact.
