# Supplementary material for: Surgical treatment of locally advanced thyroid cancer
Source: Innov Surg Sci. 2020 Sep 11;5(1-2):27–34. doi: 10.1515/iss-2020-0012 (PMC7798306; doi:10.1515/iss-2020-0012)
Supplement: Supplementary file 1 [file iss-5-20200012-s001.pdf]

Reviewer Assessment

Rudolf Roka: Surgical treatment of locally advanced thyroid Cancer

Reviewers' Comments to Original Submission

Reviewer 1: anonymous

Date received: 26-May-2020
Reviewer recommendation: **Reject for the following reasons**
Reviewer overall scoring: **Unacceptable**

Assessment Form scores: 5 = High/Yes; 3 = Medium/Adequate; 1 = Low

Is the subject area appropriate for the journal					
Does the title clearly reflect the paper's content?			3		
Does the abstract clearly reflect the paper's content					1
Do the keywords clearly reflect the paper's content?					1
Does the introduction present the problem clearly?				2	
Are the results/ conclusions justified?					1
How comprehensive and up-to-date is the subject matter presented?					1
How adequate is the data presentation?					1
Are units and terminology used correctly?				2	
Is the number of cases adequate?					1
Are the experimental methods/ clinical studies adequate?				2	
Is the length appropriate in relation to the content?					1
Does the reader get new insights from the article?					1
Please rate the practical significance.					1
Please rate the accuracy of methods.					1
Please rate the statistical evaluation and quality control.					1
Please rate the appropriateness of the figures and tables.					1
Please rate the appropriateness of the references.				2	
Please evaluate the writing style and use of language.					1
Please judge the overall scientific quality of the manuscript.					1
Are the methods used worthy of reproduction in greater deal?	No				

Comments to author: Review aiming to summarize some data from the literature concerning various aspects dealing with surgical treatment of advanced thyroid cancer. The literature compilation, however, is very selective and does not contain major studies referring outcome data concerning different cancer types, the extent of resection, and the result of resection according to the R-classification. The review is rather propedeutic, but not showing any new aspects of the subject. The tables as the list of references are insufficient, the figures are unsuitable.

Reviewer 2: anonymous

Date received: 27-Jun-2020
Reviewer recommendation: **Accept in present form**
Reviewer overall scoring: **High**

Assessment Form scores: 5 = High/Yes; 3 = Medium/Adequate; 1 = Low

Is the subject area appropriate for the journal	5				
Does the title clearly reflect the paper's content?	5				
Does the abstract clearly reflect the paper's content	5				
Do the keywords clearly reflect the paper's content?		4			
Does the introduction present the problem clearly?	5				
Are the results/ conclusions justified?	5				
How comprehensive and up-to-date is the subject matter presented?	5				
How adequate is the data presentation?	5				
Are units and terminology used correctly?	5				
Is the number of cases adequate?			3		
Are the experimental methods/ clinical studies adequate?		4			
Is the length appropriate in relation to the content?	5				
Does the reader get new insights from the article?	5				
Please rate the practical significance.	5				
Please rate the accuracy of methods.		4			
Please rate the statistical evaluation and quality control.			3		
Please rate the appropriateness of the figures and tables.	5				
Please rate the appropriateness of the references.	5				
Please evaluate the writing style and use of language.	5				
Please judge the overall scientific quality of the manuscript.	5				
Are the methods used worthy of reproduction in greater deal?					

Comments to author: The paper is an interesting review on surgical strategies in rare and complex tumour presentations and the authors may be congratulated for presenting a complex subject with evident surgical experience. Presentation, language and formatting are good. The paper may be accepted in the present form.

Reviewer 1: anonymous

Date received: 08-Jul-2020
Reviewer recommendation: **Return to author for minor modifications**
Reviewer overall scoring: **High**

Assessment Form scores: 5 = High/Yes; 3 = Medium/Adequate; 1 = Low

Is the subject area appropriate for the journal	5				
Does the title clearly reflect the paper's content?	5				
Does the abstract clearly reflect the paper's content			3		
Do the keywords clearly reflect the paper's content?			3		
Does the introduction present the problem clearly?	5				
Are the results/ conclusions justified?		4			
How comprehensive and up-to-date is the subject matter presented?			3		
How adequate is the data presentation?			3		
Are units and terminology used correctly?			3		
Is the number of cases adequate?			3		
Are the experimental methods/ clinical studies adequate?		4			
Is the length appropriate in relation to the content?		4			
Does the reader get new insights from the article?			3		
Please rate the practical significance.		4			
Please rate the accuracy of methods.		4			
Please rate the statistical evaluation and quality control.			3		
Please rate the appropriateness of the figures and tables.	5				
Please rate the appropriateness of the references.	5				
Please evaluate the writing style and use of language.		4			
Please judge the overall scientific quality of the manuscript.		4			
Are the methods used worthy of reproduction in greater deal?					

Comments to author: This is a nicely written manuscript about the surgical Management of advanced thyroid Cancer. The surgical strategies presented are not new, but the reader of the article will get substantial Information by this review. I recommend different key words for better identification of the mansucript: thyroid cancer - surgery - multiviszeral resection - reconstruction

Authors' Response to Reviewer Comments

Date received: DD-MM-2020

Response to reviewer 1

I agree that the literature compilation is very selective, but –in my opinion –in accordance to the special subjects of this paper. The major studies dealing with surgery of locally invasive thyroid cancer are collected in the two tables.

Table 1 illustrates the philosophy of experienced authors in principle (excluding those with minor case load).

Table 4 shows some aspects which have major impact for the treatment, including the type of resection, R-status and outcome. In this table I added the relevant information about the tumor pathology as described.

The main attention in this paper was given to technical details. The figures illustrate some important findings in the literature as well as some aspects of my personal experience.

Response to reviewer 2

Thank you for the good comment.

Response to reviewer 3

Thank you for the good comment. I agree with your recommendation regarding the keywords.

Comments by the Editor-in-Chief on Revised Submission

All reviewer concerns raised were addressed satisfactorily. The manuscript may be published in the present stage.
